# Risk factors associated with respiratory distress syndrome in late preterm infants

**DOI:** 10.12669/pjms.40.9.9078

**Published:** 2024-10

**Authors:** Hongbin Zhu, Yueyi Wang, Haiwei Yin, Fang Liu, Yanfei Ma, Xinyue Li

**Affiliations:** 1 Hongbin Zhu, Department of Paediatrics, Maternity & Child Care Center of Qinhuangdao, Qinhuangdao 066000, Hebei, China; 2 Yueyi Wang, Department of Paediatrics, Maternity & Child Care Center of Qinhuangdao, Qinhuangdao 066000, Hebei, China; 3 Haiwei Yin, Department of Paediatrics, Maternity & Child Care Center of Qinhuangdao, Qinhuangdao 066000, Hebei, China; 4 Fang Liu, Department of Paediatrics, Maternity & Child Care Center of Qinhuangdao, Qinhuangdao 066000, Hebei, China; 5 Yanfei Ma, Department of Paediatrics, Maternity & Child Care Center of Qinhuangdao, Qinhuangdao 066000, Hebei, China; 6 Xinyue Li, Department of Paediatrics, Maternity & Child Care Center of Qinhuangdao, Qinhuangdao 066000, Hebei, China

**Keywords:** Late Preterm Infants, Neonatal Respiratory Distress Syndrome, Risk Factors, Diagnosis

## Abstract

**Objective:**

To investigate the risk factors for neonatal respiratory distress syndrome (NRDS) in late preterm infants.

**Method:**

A retrospective analysis was performed on the clinical data of 86 late preterm infants with a gestational age of 34-36^+6^ weeks who were admitted to Maternity & Child Care Center of Qinhuangdao from June 2022 to June 2023 and with complete clinical records. All enrolled infants were divided into the non-NRDS group(*n*=51) and the NRDS group(*n*=35) according to the presence or absence of NRDS.

**Result:**

No statistically significant differences were observed in birth weight, gestational age and gravidity between the two groups(*p>*0.05), while there were statistically significant differences in fetal gender, mode of delivery and presence or absence of asphyxia(*p<*0.05). Moreover, no statistically significant differences were found in advanced maternal age, anemia, multiple births, and gestational hypertension(*p>*0.05), while there were statistically significant differences in placental abnormalities, intrauterine distress, premature rupture of membranes, and gestational diabetes mellitus(*p<*0.05). Logistic regression analysis revealed that fetal gender(male), placental abnormalities, intrauterine distress, premature rupture of membranes, and gestational diabetes mellitus were risk factors for NRDS in late preterm infants(*p<*0.05).

**Conclusion:**

Late preterm infants suffer from NRDS due to a variety of complex pathogenic causes, with numerous complications. Factors such as male fetal gender, placental abnormalities, intrauterine distress, premature rupture of membranes, and gestational diabetes mellitus may increase the risk of NRDS in late preterm infants. In clinical observation, it is necessary to strengthen monitoring efforts and take timely measures to intervene in the course of NRDS.

## INTRODUCTION

Neonatal respiratory distress syndrome (NRDS) is a lung disorder in infants that is caused by the deficiency of pulmonary surfactant (PS) or insufficient development of lung structure. It is manifested as moaning, purple skin and dyspnoea soon after birth, and may even cause respiratory failure in severe cases, which is one of the primary causes of neonatal death. Pulmonary examination of NRDS shows decreased radiolucency, reticular and granular shadows, and even white lungs, which often occur in premature infants.^1^,^2^ Late preterm infants are defined as those born at 34-36^+6^ weeks of age. Such a group covers more preterm infants, with higher gestational age and weight, and is closer to full-term infants in lung development. As a result, some families and even physicians not being sensitive enough to the early symptoms of NRDS in late preterm infants, delaying the timing of treatment. Late preterm infants experience an increased incidence of NRDS, with a gradual increase in the proportion of severe and even death.^3^ Therefore, it is of great necessity to investigate the influencing factors of NRDS in late preterm infants in order to take timely and correct treatment measures. In this study, a retrospective analysis was performed on the clinical data of 86 late preterm infants admitted to Maternity & Child Care Center of Qinhuangdao from June 2022 to June 2023, and the clinical characteristics of infants and parturients were analyzed, with a view to laying a solid data foundation for the diagnosis of NRDS in late preterm infants.

## METHODS

This was a retrospective study. Eighty-six late preterm infants with a gestational age of 34-36^+6^ weeks who were admitted to Maternity & Child Care Center of Qinhuangdao from June 2022 to June 2023 and had complete clinical records were enrolled as subjects. They were divided into the non-NRDS group(*n*=51) and the NRDS group(*n*=35) according to the presence or absence of NRDS. The diagnosis of NRDS is based on the Montreux Criteria for Neonatal Acute Respiratory Distress Syndrome established in 2017.

### Ethical Approval:

The study was approved by the Institutional Ethics Committee of Maternity & Child Care Center of Qinhuangdao (No.: QHDFY-2023031009; date: March 10, 2023), and written informed consent was obtained from all participants’ guardians.

### Study methods and statistical indicators:

The risk factors for RDS in late preterm infants were analyzed by comparing the clinical indicators between the NRDS group and the non-NRDS group. The indicators included for statistical analysis mainly include the conditions of infants and mothers. The former includes fetal gender, birth weight, gestational age, mode of delivery, gravidity, and presence of asphyxia; while the latter includes multiple births, placental abnormalities, intrauterine distress, premature rupture of membranes, advanced age, anemia, gestational hypertension, and gestational diabetes mellitus.

### Statistical analysis:

All data in this study were collated and statistically analyzed by SPSS 22.0 software. The measurement data in the table were expressed as (*χ̅*±*S*), and differences between groups were analyzed using *t* test. Count data were expressed as frequency (percentage), and the χ^2^ test was utilized to analyze the difference between groups. Multivariate logistic regression analysis was used to analyze the risk factors determined after analysis. P*<*0.05 was considered a statistically significant difference.

## RESULTS

A total of 86 late preterm infants were enrolled in the study, including 35 cases with NRDS and 51 cases without NRDS. The basic conditions of the infants in the two groups are shown in Table-I. There were statistically significant differences in fetal gender, mode of delivery and presence or absence of asphyxia between the two groups (*p<*0.05), but no statistically significant differences were observed in birth weight, gestational age and gravidity (*p>*0.05).

**Table-I:** Comparative analysis of the basic conditions of the two groups.

	NRDS group (n=35)	Non-NRDS group (n=51)	t/χ^2^	P
Gender cases (%)			5.830	0.016
Male	23 (65.71)	20 (39.22)		
Female	12 (34.29)	31 (60.78)		
Birth weight (g, *χ̅*±*S*)	2510.71±344.54	2544.82±292.34	-0.494	0.623
Gestational age (weeks, *χ̅*±*s*)	35.52±0.71	35.54±0.91	-0.079	0.937
Mode of delivery cases (%)			9.471	0.002
Cesarean section	22 (62.86)	15 (29.41)		
Vaginal delivery	13 (37.14)	36 (70.59)		
Gravidity cases (%)	1.71±0.67	1.65±0.69	0.451	0.653
Asphyxia cases (%)	11 (31.43)	7 (13.73)	3.931	0.047

The basic conditions of the infants’ mothers in the two groups are shown in Table-II. There were statistically significant differences in maternal pregnancy complications including placental abnormalities, intrauterine distress, premature rupture of membranes, and gestational diabetes mellitus between the two groups (*p<*0.05), but no statistically significant differences were found in advanced maternal age, anemia, multiple births, and gestational hypertension (*p>*0.05).

**Table-II:** Comparative analysis of the basic conditions of the infants’ mothers between the two groups cases (%).

	NRDS group (n=35)	Non-NRDS group (n=51)	χ^2^	P
Advanced age	5 (14.29)	6 (11.76)	0.118	0.731
Anemia	5 (14.29)	7 (13.73)	0.005	0.941
Placental abnormalities	14 (40.00)	8 (15.69)	6.445	0.011
Intrauterine distress	12 (34.29)	6 (11.76)	6.361	0.012
Premature rupture of membranes	13 (37.14)	8 (15.69)	5.178	0.023
Multiple births	5 (14.29)	5 (9.80)	0.406	0.524
Gestational hypertension	8 (22.86)	7 (13.73)	1.202	0.273
Gestational diabetes mellitus	15 (42.86)	7 (13.73)	9.253	0.002

Logistic regression analysis was performed for the indicators with significant differences in univariate analysis, as shown in Table-III. The results showed that fetal gender (male), placental abnormalities, intrauterine distress, premature rupture of membranes, and gestational diabetes mellitus were risk factors for NRDS in late preterm infants (*p<*0.05).

**Table-III:** Logistic regression analysis of risk factors associated with NRDS in late preterm infants.

Risk factors	β	SE	Wald	OR	P	95%CI
Gender	1.606	0.650	6.114	4.984	0.013	1.395~17.802
Mode of delivery	1.086	0.658	2.724	2.961	0.099	0.816~10.747
Asphyxia	0.604	0.795	0.578	1.829	0.447	0.385~8.682
Placental abnormalities	2.274	0.772	8.677	9.716	0.003	2.140~44.109
Intrauterine distress	2.223	0.781	8.105	9.231	0.004	1.999~42.636
Premature rupture of membranes	1.843	0.735	6.291	6.313	0.012	1.496~26.637
Gestational diabetes mellitus	2.491	0.789	9.977	12.072	0.002	2.573~56.632
Constant	-8.007	1.853	18.680	3.331E-4	<0.001	—

## DISCUSSION

As shown in this study, there were statistically significant differences in fetal gender, mode of delivery, asphyxia, placental abnormalities, intrauterine distress, premature rupture of membranes and gestational diabetes mellitus between infants with NRDS and normal late preterm infants. Logistic regression analysis revealed that fetal gender (male), placental abnormalities, intrauterine distress, premature rupture of membranes, and gestational diabetes mellitus were high risk factors for NRDS in late preterm infants, and mode of delivery (cesarean section) tended to be a risk factor for NRDS. NRDS arises from a variety of complex pathogenic causes, such as pulmonary infection, meconium aspiration, sepsis, lung failure, insufficient secretion of PS. It may give rise to symptoms such as hypothermia, renal failure, coagulation, and respiratory failure, with a severe rate as high as 55.6%.^4^ Despite saving the lives of infants with NRDS, mechanical ventilation measures can bring complications such as pneumothorax and pulmonary hemorrhage. Therefore, it is of great necessity to make early diagnosis, early observation, early detection and early treatment of NRDS in late preterm infants.

Gender (male) seems to be consistently recognized as a risk factor for NRDS. Stylianou-Riga P et al.^5^ analyzed the data of 134 newborns and showed that 74.5% of the 55 infants with NRDS were male, while only 53.6% of the healthy newborns were male, which was consistent with the results of this study. Stylianou-Riga P et al.^5^ showed that fetal gender (male) is a high-risk factor for NRDS. More studies by Seaborn T et al.^6^ pointed out that male infants have a higher incidence of NRDS than female infants, which is because the high concentration of androgen inhibits alveolar development and delays lung development in male infants, whereas estrogen promotes lung development in female infants. The effects of sex hormones on lung development are extremely complex and cannot be simply summarized as inhibition or promotion. There is an exquisite regulatory network between the two. In any case, it is at least clear that gender has a direct effect on the development of NRDS in late preterm infants, but the specific mechanism needs to be further studied.

Recent years have witnessed the gradual improvement of medical level and the maturity of cesarean section techniques, followed by an increasing number of pregnant women tending to give birth by cesarean section. Evidence suggests an association between the cesarean section and the incidence of NRDS. Li Y et al.^7^ conducted an in-depth analysis of the data of 25 NRDS based on Mata analysis. The results showed that the combined OR of cesarean section and NRDS risk was 1.76 (95%CI 1.48-2.09), that of elective cesarean section and NRDS risk was 2.38 (95%CI 1.89-2.99), and that of emergency cesarean section and NRDS risk was 1.85 (95%CI 1.34-2.56), indicating that cesarean section was associated with an increased risk of NRDS. These results are consistent with the findings of this study.

Studies have pointed out that infants born by cesarean section have a significantly higher lung fluid volume at three hours after delivery and are more difficult to respond to PS than those born by vaginal delivery.^8^ SP, as a mixture of protein and lipid, is secreted by alveolar type II epithelial cells. It is one of the main structures for the maintenance of lung function by providing pulmonary alveoli with surface activity to avoid excessive expansion and collapse during respiration.^9^ The difficulty of SP functioning leads to the difficulty of gas exchange in neonates and accelerates the process of NRDS. In addition, infants delivered by cesarean section lack uterine contraction stimulation and have lower secretion of catecholamines and glucocorticoids associated with delivery, which affects the maturation of fetal lungs.^10^ For effective ways to reduce the risk of NRDS in late preterm infants, appropriate delivery modes should be selected according to maternal conditions to avoid unnecessary cesarean section, and the cesarean section rate should be controlled.

Neonatal asphyxia, or birth asphyxia, is defined as a pathological condition in which newborns are unable to breathe normally and spontaneously after birth, causing hypoxia, hypercapnia, and multiple organ damage. It is extremely likely to cause lung. It was suggested in this study that neonatal asphyxia increases the risk of NRDS in late preterm infants. This may be explained that in the case of hypoxia caused by neonatal asphyxia, blood oxygen is mainly supplied to important organs such as the heart and brain, but not sufficiently to the lungs. As a result, the anaerobic metabolism of acidic substances increases, which damages alveolar capillary endothelial cells and type II epithelial cells.

Concurrently, alveolar capillary permeability increases, plasma proteins penetrate the alveoli, and PS secretion is inhibited so that it is unlikely to maintain alveolar tension, thereby aggravating the risk of NRDS.^11^ Ye W et al.^12^ conducted a retrospective analysis of 320 infants with NRDS in South China and pointed out that neonatal asphyxia is one of the risk factors for NRDS. Late premature infants are in the transitional stage of lung development to the alveolar stage, and will undoubtedly suffer even worse if there is asphyxia at this time. To avoid this, more intensive and frequent monitoring of late preterm infants is needed.

The placenta, as the key medium of material exchange between the fetus and its mother, plays a role in controlling the normal and healthy development of the fetus. Placental abnormalities include placental vascular abnormalities, placental inflammation, placental umbilical cord abnormalities, placental abruption and so on. These conditions pose a risk of affecting the delivery of oxygen and nutrients, causing delays in fetal growth and organ development. Maternal vascular hypoperfusion is a relatively common placental pathology. Girsch JH et al.^13^ revealed the correlation between maternal vascular hypoperfusion and increased risk of NRDS in an analysis of 990 birth cases. Abnormally invasive placenta and placenta previa will increase the probability of NRDS in newborns, especially fetuses falling into the former type need longer respiratory support time.^14^ The above findings, together with the data from this study, strongly prove that placental abnormalities and the resultant hypoxia are one of the risk factors for respiratory problems and NRDS in late preterm infants.

Intrauterine distress, as a late pregnancy complication induced by hypoxia and acidosis in the uterus due to various reasons, is characterized by frequent or decreased fetal movement and abnormal fetal heart rate. It has a high possibility of causing maternal hemorrhage and neonatal asphyxia, resulting in a great impact on the development of the fetal brain, nerves, lungs and other functions.^15^ Intrauterine distress was revealed to be an independent risk factor for neonatal NRDS in the present study. This may be because intrauterine distress leads to abnormal pulmonary blood flow, leading to lung injury and increasing the risk of NRDS in neonates. Other studies have also reached the same conclusion, showing that the combined ORs of intrauterine distress and NRDS risk are 5.59 (95%CI 3.84-8.14) and 2.427 (95%CI 1.079-5.458)^12^ respectively, significantly increasing the risk of neonatal NRDS. Intrauterine distress is often associated with asphyxia, so special attention should be paid to the history of perinatal hypoxia in late preterm infants and timely intervention measures should be taken.

Premature rupture of membranes refers to the spontaneous rupture of fetal membranes during pregnancy, which is prone to preterm birth. Fetuses born under this condition suffer from incomplete lung development and insufficient maturity, which is at high risk of respiratory problems. They are also prone to complications such as chorioamnionitis and intrauterine infection.^16^ Our study concluded that premature rupture of membranes significantly increases the risk of NRDS in late preterm infants. However, Tchirikov M et al.^17^ believed that chorioamnionitis caused by premature rupture of membranes can increase fetal interleukin-6 level, induce SP-A secretion and accelerate lung maturation, thereby reducing the risk of NRDS in preterm infants. Contrary to the views of this study, some studies have shown that the incidence of NRDS and respiratory failure in neonates without premature rupture of membranes is 20% and 40.4%, respectively, which are significantly higher than those in neonates with premature rupture of membranes.^18^

More studies have analyzed that premature rupture of membranes will increase the incidence of NRDS, which may be related to the resulting inflammatory response.^19^ The correlation between premature rupture of membranes and NRDS is still controversial. However, it is certain that premature rupture of membranes and its possible complications are closely related to fetal lung diseases. It is necessary to closely monitor premature rupture of membranes and take measures to reduce the risk of pulmonary morbidity.

Gestational diabetes mellitus, as a severe complication of pregnancy, refers to the persistent symptoms of hyperglycemia during pregnancy in pregnant women with no history of diabetes before pregnancy. It can disturb the metabolism of pregnant women and fetal development, and affect the lung maturation of newborns. It has been pointed out in many studies that gestational diabetes mellitus can increase the risk of neonatal NRDS, especially in late preterm infants greater than 34 weeks of gestation^12^, which is consistent with the results of the present study.

The pathogenesis of gestational diabetes mellitus is intricate, dominated by insulin resistance and insufficient insulin secretion. On the one hand, high levels of blood sugar in pregnant women promote fetal insulin secretion and inhibit the secretion of thyroid hormones, glucocorticoids, etc.; on the other hand, they reduce the secretion and release of SP from alveolar type II cells and delay the maturation of fetal lung surfactant system, ultimately leading to increased risk of lung diseases such as NRSD. Contrary to this, some studies have suggested that gestational diabetes mellitus itself is not the main factor leading to NRDS in late preterm infants.^20^ But in any case, much attention should still be paid to gestational diabetes mellitus and its complications.

### Limitations of this study:

However, the limitation of this study is that only a part of the cases were analyzed retrospectively. In future more cases should be included for further analysis, and the clinical manifestations of NRDS should be deeply analyzed from multiple perspectives, in order to establish a systematic and comprehensive prediction system of NRDS and provide a theoretical basis for the prevention of NRDS.

## CONCLUSIONS

Fetal gender (male), asphyxia, placental abnormalities, intrauterine distress, premature rupture of membranes, and gestational diabetes mellitus may be risk factors for NRDS in late preterm infants, and cesarean section has a tendency to become a risk factor for NRDS. However, NRDS in late preterm infants is often not caused by a single factor, but a complex pathogenic cause with many complications. All of these take a heavy toll on the bodies of newborns and the psychological conditions of their parents.

### Authors’ Contributions:

**HZ** and **YW:** Carried out the studies, participated in collecting data, and drafted the manuscript, and are responsible and accountable for the accuracy or integrity of the work.

**HY** and **FL:** Performed the statistical analysis and participated in its design.

**YM** and **XL:** Performed the statistical analysis and participated in its design.

All authors read and approved the final manuscript.
